# Detecting disease-associated genes with confounding variable adjustment and the impact on genomic meta-analysis: With application to major depressive disorder

**DOI:** 10.1186/1471-2105-13-52

**Published:** 2012-03-29

**Authors:** Xingbin Wang, Yan Lin, Chi Song, Etienne Sibille, George C Tseng

**Affiliations:** 1 Department of Biostatistics, University of Pittsburgh, Pittsburgh, PA 15261, USA; 2 Department of Psychiatry, University of Pittsburgh, Pittsburgh, PA 15260, USA; 3 Department of Human Genetics, University of Pittsburgh, Pittsburgh, PA 15261, USA; 4 Department of Computational and Systems Biology, University of Pittsburgh, Pittsburgh, PA 15260, USA

## Abstract

**Background:**

Detecting candidate markers in transcriptomic studies often encounters difficulties in complex diseases, particularly when overall signals are weak and sample size is small. Covariates including demographic, clinical and technical variables are often confounded with the underlying disease effects, which further hampers accurate biomarker detection. Our motivating example came from an analysis of five microarray studies in major depressive disorder (MDD), a heterogeneous psychiatric illness with mostly uncharacterized genetic mechanisms.

**Results:**

We applied a random intercept model to account for confounding variables and case-control paired design. A variable selection scheme was developed to determine the effective confounders in each gene. Meta-analysis methods were used to integrate information from five studies and post hoc analyses enhanced biological interpretations. Simulations and application results showed that the adjustment for confounding variables and meta-analysis improved detection of biomarkers and associated pathways.

**Conclusions:**

The proposed framework simultaneously considers correction for confounding variables, selection of effective confounders, random effects from paired design and integration by meta-analysis. The approach improved disease-related biomarker and pathway detection, which greatly enhanced understanding of MDD neurobiology. The statistical framework can be applied to similar experimental design encountered in other complex and heterogeneous diseases.

## Background

Microarray experiment enables researchers to examine the expression of thousands of genes in parallel. Differentially expressed (DE) gene detection is one of the most common analyses in microarray. In such an analysis, genes differentially expressed under multiple conditions are detected and are used for generating further biological hypotheses, developing potential diagnostic tools, or investigating therapeutic targets. The extensive applications of microarray technology have led to an explosion of gene expression profiling studies publicly available. However, the noisy nature of microarray data, together with small sample size in each study, often results in inconsistent biological conclusions [1-3]. Therefore, meta-analysis, a set of statistical techniques to combine multiple studies under related research hypotheses, has been widely applied to microarray analysis to increase the reliability and robustness of results from individual studies. In the literature, three major categories of meta-analysis methods have been applied to genomic meta-analysis: combining effect sizes [4,5], combining p-values [6-8] and combining rank statistics [9,10]. In general, different approaches have different underlying assumptions and pros and cons in the applications [11,12].

Major depressive disorder (MDD) is a heterogeneous illness with mostly uncharacterized pathology. Despite several gene expression studies of MDD [13-17] published, the biological mechanisms of MDD remain mostly uncharacterized [18]. Although biomarkers and pathways have been identified in specific studies, the findings are not consistently observed from study to study. This variability may be due to several factors. Firstly, MDD is thought to be a complex and heterogeneous disease [19], associated with multiple genetic, post-translational, and environmental factors. Furthermore, patients might have varying disease severity, with some having psychotic features as well as exposure to a variety of medications and dosage levels to control their illness. Secondly, the genetic disease effects are potentially confounded by many covariates, which include (1) demographic variables such as age, gender and race; (2) clinical variables such as anti-depressant drug usage, death by suicide and alcohol dependence; (3) technical variables inherent in the use of post-mortem brain samples, such as the pH level of brain tissues, brain region and post-mortem interval (PMI). In statistical terms, confounding variables are defined as extraneous variables that can adversely affect the relationship between the independent variable (i.e. disease state) and dependent variable (i.e. gene expression). If the statistical models employed to identify differentially expressed genes fail to incorporate these sources of heterogeneity (potential confounding variables), not only can this reduce the statistical power, but also it will introduce sources of spurious signals to the gene detection. Finally, sample sizes for these studies are generally small (between 10-25 pairs of MDDs and controls) due to the limited availability of suitable brain specimens and the significant costs associated with their collection.

In this paper, we propose a statistical framework to tackle overall weak signal expression profiles in MDD that have small sample size, case-control paired design and confounding covariates in each study. We use a set of five MDD expression profiles as an illustrative example. In the literature, most analyses of similar data structure either ignored the potentially confounding covariates by using paired or unpaired t-test [16,20,21] or applied simple linear regression model to incorporate all covariates [22,23]. The former approach undoubtedly ignored effects from confounding covariates; the latter approach was not efficient or even not applicable when the number of covariates is large and the number of samples in each study is small. In this paper, we will propose a framework that uses a random intercept model (RIM) to account for the case-control paired design and confounding covariates in single study analysis. An improved RIM with novel gene-specific variable selection (namely RIM_minP or RIM_BIC to be introduced later) will be performed to accommodate the small sample size and relatively large number of covariates in individual studies. We will then apply and compare two popular meta-analysis methods: Fisher's method [24,25] and maximum p-value method [26-28]. The application of this approach to combine five MDD microarray studies show improved DE gene detection competency and superior statistical significance of pathway detection using our proposed method. Simulations considering various correlation structures among disease state, gene expression and covariates will be performed to demonstrate better performance of this framework. Our proposed framework is general and applicable in commonly encountered microarray meta-analysis of complex genetic diseases.

## Methods

### Description of motivating MDD data

This research is motivated from the meta-analysis of combining five MDD transcriptomic studies. Brain tissues of three patient cohorts (MD1, MD2 and MD3) obtained from different sources at different time were analyzed. For all three patient cohorts, tissues from the anterior cingulated cortex (ACC) brain region were analyzed by microarray experiments independently to generate three microarray studies: MD1_ACC, MD2_ACC and MD3_ACC. Similarly, tissues from the amygdala (AMY) brain region in MD1 and MD3 cohorts were analyzed to generate MD1_AMY and MD3_AMY. Details of the five patient cohorts and microarray studies are available in Additional file 1: Table S1. In each patient cohort, MDD patients were matched to control patients by three demographic variables: age, sex and race. Case-control paired design is proven to increase statistical power significantly, especially for expensive experiments that inhibit large sample size. This is exactly the case in most brain disorder studies using post-mortem samples. Three additional clinical variables (alcohol dependence, evidence of taking anti-depressant drugs and death by suicide) and two technical variables (pH level of brain tissues and post-mortem interval PMI) were also available for each patient. Among the covariates described above, six variables (age, alcohol, drug, suicide, pH and PMI) were considered potential confounders in the DE gene detection of MDD. These six covariates were not highly correlated with each other in our analysis and thus the collinearity issue does not exist in the linear models below (see Additional file 1: Table S2).

### Data pre-processing, gene matching and gene filtering

Microarray images were scanned and summarized by manufacturers' defaults. Data from Affymetrix arrays were processed by RMA method and data from Illumina were processed by manufacturer's software for probe analysis. When samples in each study were processed in multiple batches, potential batch effects were evaluated and samples were normalized to have the same sample means and sample standard deviations to correct batch biases when necessary. Probes (or probe sets) were then matched to official gene symbols using Bioconductor packages. When multiple probes (or probe sets) matched to an identical gene symbol, the probe that presented the greatest inter-quartile range (IQR) was selected to represent the target gene symbol. Larger IQR represents greater variability (and thus greater information content) in the data and this probe matching method has been recommended in Bioconductor [29]. After genes were matched across five studies, 16,715 unique gene symbols were available across all five studies and intensities were all log-transformed (base 2). Two sequential steps of gene filtering were then performed. In the first step, we filtered out genes with very low gene expression that were identified with small average expression values across majority of studies. Specifically, mean intensities of each gene across all samples in each study were calculated and the corresponding ranks were obtained. The sum of such ranks across five studies of each gene was calculated and genes with the lowest 30% rank sum were considered un-expressed genes (i.e. small expression intensities) and were filtered out. Similarly, in the second step, we filtered out non-informative (small variation) genes by replacing mean intensity in the first step with standard deviation. Genes with the lowest 40% rank sum of standard deviations were filtered out. Additional file 1: Figure S1 shows the pre-processing diagram and the number of genes remained in each pre-processing step. Finally, 7,020 matched genes (16715 × 0.7 × 0.6 = 7020) in five studies were analysed. We note that the somewhat ad hoc gene filtering procedure is necessary and is commonly used in microarray analysis. Although it runs the risk to ignore important DE genes, it has benefits of reducing false positives from non-expressed or non-informative genes and increasing statistical power in multiple comparison procedure. Filtering down to ~7000 genes is moderate and adequate since the size usually keeps majority of acting genes of the genome in the analysis.

### Single study analysis for DE gene detection

Paired t-test Paired t-test was performed as a comparison to the method we developed below. This method took into the MDD and control paired design into consideration but ignored the confounding covariates. We also tested non-parametric Wilcoxon signed rank test. The results were similar to paired t-test and thus not shown in the result.

Random intercept model (RIM) and fixed effects model (FEM) To account for paired design (MDD samples paired with corresponding controls) and existence of MDD related covariates, we applied a random intercept model (RIM). For a given gene *g*, we fit the model

$$Y_{gik}=\mu_{g}+\beta_{g0}X_{0ik}+\sum_{l=1}^{L}\beta_{gl}X_{lik}+\alpha_{k}+\varepsilon_{gik}$$

In the model, *Y_gik _*was the gene expression value of gene *g *(1 ≤ *g *≤ *G*) and disease status *i *(*i *= 1 for control and 2 for MDD) in pair *k *(1 ≤ *k *≤ *K*). *X_0ik _*was the disease label that took value one if the sample was MDD and zero if the sample was a control. *X_lik _*represented values for potential confounding covariate *l *(1 ≤ *l *≤ 6; 0-1 binary variables for alcohol, drug and suicide; numerical variables for age, pH and PMI). *α_k _*was the random intercept from a normal distribution with mean zero and variance *τ_g_^2^*, which represented the deviation of averaged expression values in the *k*^th ^pair from the average in the whole population. Finally, *ε_gik _*were independent random noises that followed a normal distribution with mean zero and variance *σ_g_^2^*. Under this model, *β_g0 _*was the disease effect of gene *g *and was the parameter of major interest. To obtain an MDD-associated biomarker candidate list in a single study analysis, likelihood ratio test (LRT) was used to calculate the *p*-values of testing H_0_: *β_g0 _*= 0 (vs H_A_: *β_g0 _*≠ 0). We denote this method as RIM_ALL (random intercept model containing all covariates) as opposed to the models with variable selection in the next section. The p-values from RIM_ALL were then corrected by Benjamini-Hochberg procedure [30] for multiple comparison to control false discovery rate (using "p.adjust" function in R).

Fixed effects model (FEM) below ignores the paired design while still considers the covariates in the model. It can be used when diseased and control samples are not paired. We used it to compare with RIM to evaluate the impact of case-control design.

$$Y_{gik}=\mu_{g}+\beta_{g0}X_{0ik}+\sum_{l=1}^{L}\beta_{gl}X_{lik}+\varepsilon_{gik}$$

RIM and FEM with variable selection Although RIM model can effectively adjust for confounding covariates in DE gene detection, the small sample size (10-25 pairs) and relatively high number of potential confounders (6 covariates) can make the model inefficient and impractical. In this paper, we developed and evaluated two choices of variable selection procedures in the random intercept model (namely, RIM_BIC and RIM_minP). Specifically, all possible RIM models that included at most two (0, 1 or 2) clinical variables were computed and compared. In RIM_BIC, the model with the smallest Bayesian Information Criterion (BIC) [31] value was selected. For RIM_minP, we selected the model that yielded the smallest p-value associated with the likelihood ratio test for testing the disease effect H_0_: *β_g0 _*= 0. Conceptually, BIC selected the model with the best overall model fitting and prediction while minP focused on the model that gave the best statistical significance of the disease effect *β_g0_*. This additional variable selection avoided to include more than 2 clinical variables in the model and allowed assessment of biomarkers affected by different sets of covariates in each gene (e.g. disease effect of gene A may be confounded by alcohol and age while gene B may be confounded by antidepressant drug), which biologically gave more appealing interpretations and conclusions. Similar to RIM model, the likelihood ratio test was used to generate p-values of testing H_0_: *β_g0 _*= 0 in each gene for the selected model by BIC or minP. The resulting *p*-values from the LRT were, however, biased from the variable selection procedure and the type I error control was invalid. As a result, we performed a permutation test that randomly permuted the disease labels within each case-control pair to generate a null distribution for p-value assessment. Additional file 1: Figure S2 shows the simulated null distribution from permutation analysis. The skewed distribution deviating from uniform distribution between 0 and 1 showed the need of the permutation analysis for p-value correction. Similar to RIM_ALL, the p-values from RIM_minP and RIM_BIC are corrected by Benjamini-Hochberg procedure for multiple comparisons separately. Detailed algorithm of the permutation analysis and inference is described in Additional file 1: Part I.

Testing significance of interaction terms of each covariate: In the literature, age as well as other covariates has been found to be confounders of the disease effect with significant interaction term in some biomarkers [32,33]. In other words, the disease effect on gene expression may be affected by age differently in older and in younger cohorts. To evaluate the overall impact of the interaction terms in each covariate, we performed the following simple linear model

$$Y_{gik}=\mu_{g}+\beta_{g0}X_{0ik}+\beta_{gl}X_{lik}+\gamma_{gl}X_{0ik}X_{lik}+\varepsilon_{gik}$$

and random intercept model

$$Y_{gik}=\mu_{g}+\beta_{g0}X_{0ik}+\beta_{gl}X_{lik}+\gamma_{gl}X_{0ik}X_{lik}+\alpha_{k}+\varepsilon_{gik}$$

where the notations were the same as in the FEM model and RIM model but now with only one covariate (i.e. a given variable *l*) included and a corresponding interaction term involved. We performed likelihood ratio test for H_0_: *γ_gl _*= 0 to test the statistical significance of the interaction term of gene *g *and covariate *l*. Additional file 1: Table S3 summarizes the number of significant interaction terms in the genome of each covariate. The result shows that the interaction terms between each covariate (Age, pH or PMI) and MDD disease effect were not significant in most of the genes under false discovery rate FDR = 5% (Benjamini-Hochberg correction). As a result, we did not consider the interaction terms in our models throughout this paper.

### Meta-analysis for DE gene detection

Among the many microarray meta-analysis methods used in the literature, all methods have their pros and cons depending on the data structure and biological goal [11,34]. According to Birnbaum [35], Li and Tseng [36], and Tseng et al. [12], meta-analysis methods can be categorized into two types: one detects gene markers differentially expressed "in one or more studies" and the other detects genes differentially expressed in "all studies". Fisher's method and maxP method are two popular methods in the two categories, respectively, and we will apply both methods in this paper. Fisher's method calculates the sum of log-transformed *p*-vales in the meta-analysis: *V_g_^Fisher ^*= - 2Σ*_k_^K ^*= _1_log(*Pgk*) where *K *is the number of studies combined and *P_gk _*is the *p*-value of gene *g *and study *k*. Assuming independence among studies and that the model to derive *p*-values is correctly specified, *V*_g_*Fisher *follows a chi-squared distribution with degree of freedom *2K *under the null hypothesis. In contrast, the maxP method combines *p*-values by taking the maximum of *p*-values across studies: *V_g_^maxP^*= max_1 ≤ k ≤ kPgk_*V_g_^maxP^*. It can be show that *V_g_^maxP ^*follows a beta distribution with shape parameters *K *and 1 under the null hypothesis. Note that in Fisher's method, an extremely small *p*-value of one study can drive strong statistical significance of that gene no matter whether *p*-values of the other studies are small or not. On the other hand, the maxP method requires small *p*-values of all studies for a gene to be detected.

Although the parametric inference by chi-squared and beta distributions is convenient, we chose to perform permutation analysis for the inference to avoid violation of underlying assumptions. We also noted that two pairs of studies (MD1_ACC and MD1_AMY, MD3_ACC and MD3_AMY) used the same cohorts with different brain regions. The studies may be dependent from each other. To account for the dependence structure among studies from the same patients, we kept the same permutation order for each pair of studies of the same cohort in the permutation analysis of individual studies. Benjamini-Hochberg procedure implemented in R is then used to control false discovery rate.

### Evaluation by detection compentency and pathway analysis

For evaluation purpose, we compared three single-study DE gene detection methods (PT, RIM_minP and RIM_BIC). Each method was applied to five single studies and then the five single study results were combined by Fisher and maxP meta-analysis methods. As a result, a total of 3 × 7 = 21 DE gene detection results were generated and compared. To assess performance of these different methods, we applied two evaluation criteria. The first criterion compared the numbers of detected DE genes from different methods under different *p*-value thresholds using detection competency curves (x-axis: *p*-value threshold; y-axis: number of detected DE genes). This first criterion is a reflection of detection competency of different methods.

The detection competency curves alone, however, do not guarantee an improved biological finding. Thus, we evaluated the biological associations of detected DE genes from different methods with existing pathway knowledge databases in the second criterion. We collected 2,287 pathways from MsigDB [37]http://www.broadinstitute.org/gsea/msigdb/; 1,454 pathways from Gene ontology, 217 from Biocarta, 186 from KEGG and 430 from Reactome). Kolmogorov-Smirnov (KS) test was applied for each DE gene result and each pathway for pathway analysis (a.k.a. gene set analysis) [27,37]. KS test improved a commonly used Fisher's exact test in that it does not arbitrarily apply a DE gene cut-off but directly evaluate the DE evidence ordering in the genome. For the DE detection result of a given method, a smaller *p*-value from KS test reflects a stronger biological validation of the detected DE genes. The detailed KS test algorithm and inference are described in the Additional file 1: Part II.

Since 2,287 pathways were tested, aggregating the total information to conclude one method being better than the other was not a trivial task. We denote by *p_rm _*the pathway enrichment p-value of method *m *for pathway *r*. Since the majority of the 2,287 pathways were irrelevant to MDD, we first identified the top *V *most "disease-related" pathways by committee decision of the 21 DE gene detection results under comparison. Specifically, disease relatedness of a pathway *r *in method *m *was derived as the rank of *p*-values $s_{r}^{\left(m\right)}=rank_{r}\left(p_{rm}\right)$. The disease relatedness score of pathway *r *was then defined as $S_{r}^{}=\sum_{m=1}^{M}s_{r}^{\left(m\right)}$, where *M *= 21 was the total number of DE gene detection methods under comparison. A small *S_r _*reflected an overall significant pathway enrichment p-value for pathway *r *in the 21 DE gene detection results and thus was believed to be a disease related pathway. The *V *pathways with the smallest *S_r _*are selected as surrogates of "gold standard" disease-related pathways for evaluation purpose. For any two selected DE gene detection results, Wilcoxon signed rank test can be used to compare pathway enrichment *p*-values of the *V *selected pathways to determine if one method is statistically better than the other method, in terms of association with the *V *disease-related pathways. In this paper, we use *V *= 100.

### Post hoc analysis on confounding variables after meta-analysis

An important advantage of the variable selection scheme is the availability of post hoc analysis to compare selected confounders across studies. Three questions can be explored and answered: (1) Which variable(s) is the most or least frequently included in the model selection to confound with disease effect? (2) Are variables repeatedly selected across studies more frequently than by chance (e.g. alcohol is selected in most or all studies in a given gene)? (3) Are the directions of effect sizes of a variable consistent across studies (e.g. alcohol-dependent patients have higher expression than non-alcohol-dependent in most studies for a given gene)?

For the first question, we first generated a list of DE genes under a given FDR threshold and counted the frequency of each variable being selected in the gene list. The variables were ranked according to the frequencies in each study and a rank average of each variable was calculated across five studies. A small averaged rank of a covariate showed frequent appearance of the variable in the model selection and thus a frequent confounder.

For question (2), we computed a pair-wise co-appearance score (*T_1_*) for a given gene set and assessed its statistical significance. For example, gene VGF in Table 1 had detected age effects in 2 studies, alcohol effects in 3 studies, anti-depressant effect in 1 study and suicide effect in 3 studies using RIM_minP variable selection. By summing up co-appearing pairs of the five studies in each variable, we obtained a *T_1, g _*statistics of 7 $\left(C_{2}^{2}+C_{2}^{3}+0+C_{2}^{3}=7\right)$ for *g *= VGF where *C_2_^k ^*is the binomial coefficient for the number of pairs out of k elements. Summing up all 9 genes, we obtained $T_{1}=\sum_{g}T_{1,g}=60$. Permutation test was then performed to assess the statistical significance of *T_1_*.

**Table 1:** The direction of covariates effect in RIM_minP (Table 1A) and RIM_BIC (Table 1B) models for 9 MDD related genes selected from the literature

S	Age					Alcohol					Antidep					pH					PMI					Suicide					Co-appearance T_1_	Concordance T_2_	Ratio R = T_2_/T_1_
	**A**	**B**	**C**	**D**	**E**	**A**	**B**	**C**	**D**	**E**	**A**	**B**	**C**	**D**	**E**	**A**	**B**	**C**	**D**	**E**	**A**	**B**	**C**	**D**	**E**	**A**	**B**	**C**	**D**	**E**			

VGF	0	0	-1	0	-1	1	1	0	1	0	1	0	0	0	0	0	0	0	0	0	0	0	0	0	0	0	1	1	0	1	7	7	
	
SST	0	0	-1	0	-1	1	1	0	1	0	0	-1	0	0	0	1	0	0	0	0	0	0	0	0	0	0	0	1	1	1	7	7	
	
CNP	0	1	0	1	0	-1	0	0	1	0	-1	0	1	0	-1	0	-1	0	0	0	0	0	0	0	-1	0	0	1	0	0	5	2	
	
NPY	0	-1	-1	0	-1	1	0	0	1	0	0	0	0	0	0	1	0	0	0	0	0	0	0	0	0	0	-1	1	1	1	10	7	
	
TAC1	0	-1	-1	0	-1	1	0	0	1	0	1	0	0	0	0	0	0	0	1	0	0	0	0	0	0	0	-1	1	0	1	7	5	
	
MBP	0	0	0	1	1	0	-1	0	1	0	-1	0	0	0	0	0	-1	0	0	0	0	0	1	0	0	-S1	0	1	0	-1	5	2	
	
MOBP	0	0	0	0	1	0	-1	1	0	0	-1	0	0	-1	0	0	0	0	0	0	0	-1	0	0	0	-1	0	1	-1	-1	8	4	
	
RGS4	-1	0	0	0	-1	0	1	-1	1	0	1	0	-1	0	0	0	1	0	0	0	0	0	0	0	0	0	0	0	1	1	6	3	
	
HTR2A	0	-1	0	1	0	0	1	0	0	-1	-1	0	-1	0	-1	0	0	0	1	0	1	0	0	0	0	0	0	-1	0	0	5	3	

																												Total			T_1 _= 60	T_2 _= 40	R = T_2_/T_1 _= 0.67
																												
																												p-value			0.39		0.014

	Age					Alcohol					Antidep					pH					PMI					Suicide					Co-appearance T_1_	Concordance T_2_	Ratio R = T_2_/T_1_

	A	B	C	D	E	A	B	C	D	E	A	B	C	D	E	A	B	C	D	E	A	B	C	D	E	A	B	C	D	E	1	1	
VGF	0	0	-1	0	0	0	1	0	0	0	0	0	0	0	0	0	0	0	0	0	0	0	0	0	0	0	1	1	0	0	7	7	

SST	-1	0	-1	-1	-1	0	1	0	0	0	0	-1	0	0	0	0	0	0	0	0	0	0	0	0	0	0	0	1	0	1	3	3	

CNP	1	0	0	1	1	0	0	0	0	0	-1	0	0	0	0	0	0	0	0	0	0	0	0	0	-1	0	0	0	-1	0	7	7	

NPY	-1	0	-1	-1	-1	0	0	0	0	0	0	0	0	0	0	0	1	1	0	0	0	0	0	0	0	0	0	0	0	1	6	6	

TAC1	-1	0	-1	-1	-1	0	0	0	0	0	0	0	0	0	0	0	1	0	0	0	0	0	0	0	0	0	0	0	0	0	1	1	

MBP	0	0	0	1	1	0	0	0	0	0	-1	0	0	0	0	0	0	0	0	0	0	0	0	0	0	0	0	0	0	-1	3	3	

MOBP	1	0	0	1	1	0	0	0	0	0	-1	0	0	0	0	0	0	0	0	0	0	0	0	0	0	0	0	0	0	-1	3	3	

RGS4	-1	0	0	-1	-1	0	0	0	0	0	0	0	0	0	0	0	0	0	0	0	0	0	0	0	0	0	0	0	0	1	1	1	

HTR2A	0	0	0	1	0	0	1	0	0	0	0	-1	-1	0	0	0	0	-1	0	0	0	0	0	0	0	0	0	0	0	0	1	1	

																												Total			T_1 _= 32	T_2 _= 32	R = T_2_/T_1 _= 1
																												
																												p-value			0.011		0.005

* 0: variable not included in the model; 1: appear in the model with positive effect size; -1: appear in the model with negative effect size.

A: MD1_ACC, B: MD2_ACC, C: MD3_ACC, D: MD1_AMY, E: MD3_AMY

To answer question (3), we further computed rate of expression concordance among all co-appearance pairs. Specifically, we examined all co-appearing pairs that contributed to *T_1 _*and count the number of pairs that are concordant (up-regulation in both studies or down-regulation in both studies). The total aggregated score for pair-wise concordance was denoted as *T_2 _*and the ratio of concordance was *R*= *T_2_*/*T_1_*. In the example of Table 2, 40 (*T_2_*) out of 60 (*T_1_*) co-appearing pairs were concordant and *R *= 0.67. Similarly, permutation test was performed to assess the statistical significance of observed R scores. Detailed mathematical notation and permutation algorithm are outlined in Additional file 1: Part III.

**Table 2:** Number of detected DE genes using different single study analysis methods (PT, RIM_ALL, RIM_minP and RIM_BIC) in the five individual studies and by two meta-analysis methods (Fisher and maxP)

method	FDR	Individual analysis					Meta-analysis (3ACC+2AMY)	
		
		MD1_ACC	MD2_ACC	MD3_ACC	MD1_AMY	MD3_AMY	Fisher	maxP
RIM_minP	FDR = 0.05	0	0	2	0	0	0	0
	
	FDR = 0.1	0	0	2	0	725	109	99
	
	FDR = 0.15	0	0	5	0	1442	810	683

RIM_BIC	FDR = 0.05	0	0	0	0	101	0	0
	
	FDR = 0.1	0	0	1	0	506	0	0
	
	FDR = 0.15	0	0	6	0	873	38	0

RIM_ALL	FDR = 0.05	0	0	0	0	0	0	1
	
	FDR = 0.1	0	3	0	0	1	0	1
	
	FDR = 0.15	0	3	1	0	1	0	1

PT	FDR = 0.05	0	0	0	0	0	0	0
	
	FDR = 0.1	0	0	0	0	0	0	0
	
	FDR = 0.15	1	1	0	0	0	0	0

Three FDR thresholds are used (5%, 10% and 15%)

### Simulation

**Figure 1:**
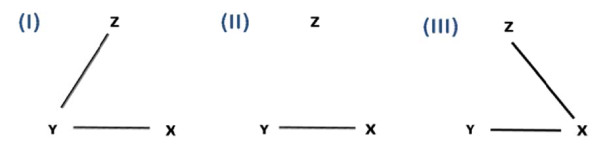
**Three correlation structures of interest among disease variables X, gene expression variable Y and putative confounding covariates Z that are used in the simulation**. Scenario I: gene expression depends on both disease state and covariates. Scenario II: gene expression depends only on disease state. Scenario III: gene expression depends on disease state directly and depends on covariates indirectly through disease state.

In the first evaluation criterion previously described, we implicated that with adequate modelling and multiple comparison correction, detecting more DE genes showed better statistical power of a method and should be a preferred method. There was, however, no rigorous proof that detecting more DE genes guaranteed better performance of a method, in terms of its type I error control and statistical power. Since the type I error and statistical power could not be evaluated in real data analysis, we performed extensive simulations below to facilitate the evaluation. For a given gene, we considered three variables: one continuous variable of gene expression Y, a corresponding binary variable of disease state X and ten variables of potential binary confounding covariate Z (Z = (*z_1_*,...,*z_10_*)). Figure 1 shows three correlation structures of interest among (X, Y, Z) that are simulated. Scenario I demonstrated that both disease state X and the first two confounding variables in Z (*z_1 _*and *z_2_*) affect gene expression, a model of most interest in this paper. Scenario II and III showed situations when confounding variables Z did not directly affect gene expression Y. In these latter two scenarios, including confounding variables Y in the model should not improve performance. The detailed simulation scheme and evaluation criteria are available in the Additional file 1: Part IV. For each scenario, we simulated a data set with 1000 independent genes and 50 samples (25 diseased and 25 controls). Among the 1000 genes, 100 are true DE genes and 900 are non-DE genes. t-test, FEM_minP, FEM_BIC and FEM_ALL were applied to evaluate the effect of modelling confounding variables and variable selection in each correlation structure. We repeated the simulation 50 times. Type I error and statistical power were calculated for each method in each data set and averaged over 50 repeated simulations. By definition, the type I error was calculated as the average number of genes detected among the 900 non-DE genes. Conversely, the statistical power was obtained as the average number of genes detected among the 100 true DE genes. Note that for simplicity, we ignored paired design in the simulation and thus applied FEM instead of RIM. Through the simulation, we expect to answer whether including confounding variables improves statistical power (t-test versus FEM_minP, FEM_BIC and FEM_ALL) and whether variable selection in the model improves power (FEM_minP and FEM_BIC versus FEM_ALL) under different scenarios.

## Results

### Recommended statistical framework

**Figure 2:**
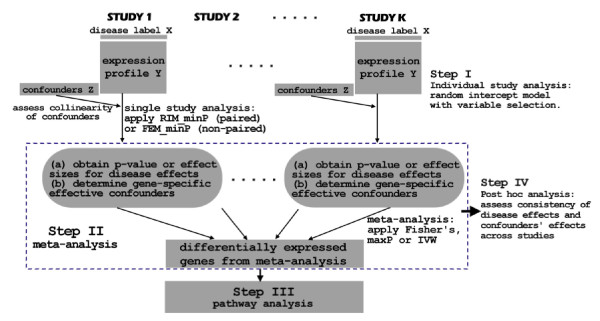
**An illustrative diagram of the proposed statistical framework**.

From the motivating MDD example, we showed in Figure 2 a diagram of the statistical framework to consider potential confounding covariates, paired design and gene-specific variable selection in the meta-analysis modelling. The framework consisted of four major steps: individual study analysis, meta-analysis, pathway analysis and post hoc analysis. In the first "individual study analysis" step, collinearity of confounders was assessed and RIM_minP or FEM_minP method with variable selection was applied depending on paired or un-paired design. One or multiple meta-analysis methods were applied and compared in Step II. Pathway analysis was then performed on the detected DE gene list(s) to identify enriched pathways in Step III. Finally, post hoc analysis was performed to summarize importance of each confounding variables and to evaluate the consistency of disease effects and confounders' effects across studies. This framework is general and applicable to any multi-study weak-signal data from a complex disease similar to the motivating MDD example.

### Comparison of various methods in single study analysis

**Figure 3:**
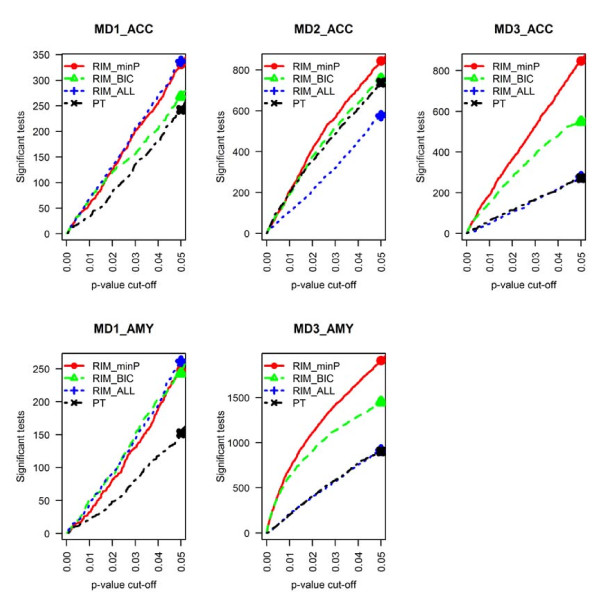
**Comparison of number of detected DE genes in individual study analyses of RIM_minP, RIM_BIC, RIM_ALL, and PT**. The result showed that RIM_minP detected the largest number of DE genes among the four methods.

Confounder adjustment and variable selection to improve DE gene detection For each single study analysis, we compared the number of detected DE genes under different p-value thresholds (*p *= 0.001, 0.005, 0.01 and 0.05) from different methods. In Figure 3, RIM_minP and RIM_BIC detected more DE genes than RIM_ALL in most studies, showing the fact that variable selection helped to ignore irrelevant clinical variables when sample size was small. Among the two variable selection methods, RIM_minP detected more genes than RIM_BIC, supporting that the focus of RIM_minP to obtain the most significant disease effect outperformed RIM_BIC's focus for best model fitting in this MDD example. Under p = 0.005, RIM_minP detected (0.8 to 1.3) times of DE genes than RIM_BIC and (0.8 to 5.5) times than RIM_ALL. The result suggested that RIM_minP is the most effective method in this data set to incorporate confounding variables in the model. In Figure 3, RIM_minP and RIM_BIC were also compared to paired t-test (PT) and were found to detect more DE genes, showing the advantage of incorporating confounding covariates in the model. RIM_minP identified (0.9 to 4.6) times DE genes than PT and RIM_BIC identified (0.8 to 4.4) times DE genes than PT under *p *= 0.005.

**Figure 4:**
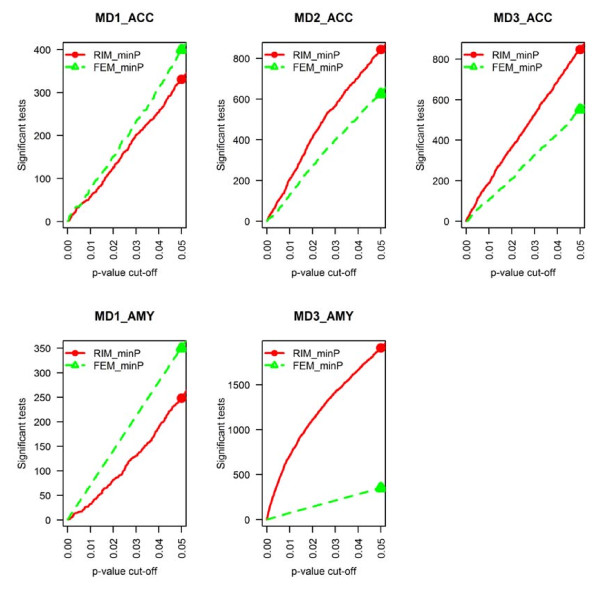
**Comparison of number of detected DE genes in individual study analyses of RIM_minP and FEM_minP**. The result showed that RIM_minP usually detected more DE genes.

Paired design to improve DE gene detection To evaluate the improvement of including paired design in the model, we compared RIM_minP and FEM_minP in Figure 4. We observed increased DE gene detection competency of RIM_minP compared to FEM_minP in three studies (MD2_ACC, MD3_AMY and MD3_ACC) but not in the other two studies (MD1_ACC and MD1_AMY). The result showed that pairing cases to controls by age, race and sex often helped increase statistical power but not always.

Conclusion Incorporation of potential confounding covariates with variable selection and considering paired design in the model of single study analysis generally increased the detection competency of disease related biomarkers.

### Comparing individual study analysis and meta-analysis

Smaller sample size in each study often results in a smaller statistical power of DE gene detection. In Table 2, the first five columns show the number of biomarkers detected by RIM_minP, RIM_BIC and PT under different false discovery rate (FDR) thresholds. After multiple comparison correction by Benjamini-Hochberg procedure, the first four single study analyses detected almost none DE genes under FDR = 5, 10 or 15%. This motivated us to perform meta-analysis to increase statistical power and provide validated findings on DE gene detection. In Table 2, the last two columns show the number of biomarkers detected by Fisher method and maxP method, respectively. The two meta-analysis methods detected more biomarkers than individual study analysis based on RIM_minP except for MD3_AMY.

**Figure 5:**
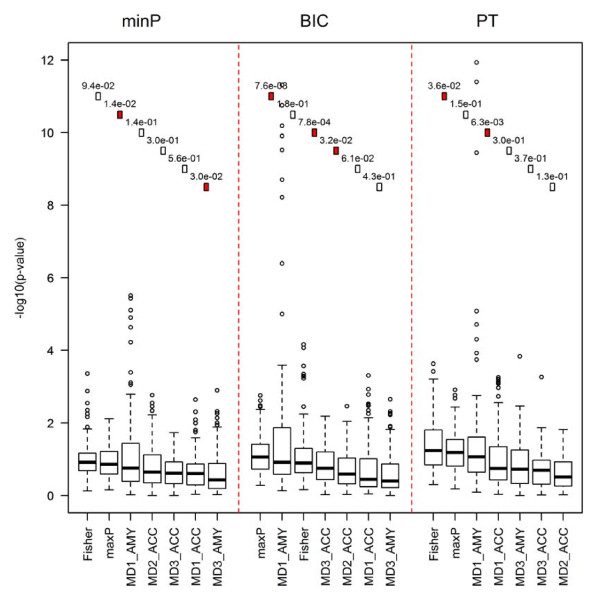
**Comparison of meta-analyse and individual analysis based on pathway analysis criterion across RIM_minP, RIM_BIC and paired t-test**. The results showed that meta-analysis produced DE analysis results with stronger association with the top 100 disease-related surrogate pathways.

To further evaluate the biological implication of the detected DE genes by various methods, pathway analysis was performed. Figure 5 showed boxplots of the minus log-transformed p-values (base 10) from pathway analysis in the top 100 disease-related surrogate pathways. DE gene detection methods were ordered by the median of the log-transformed p-values in the plot. The squares and numbers in the upper part of the figure demonstrate the p-values from Wilcoxon signed-rank test when comparing two neighbouring DE gene detection methods (numbers show the p-values and filled squares represent that the corresponding p-value is smaller than 0.05). The result showed a clear pattern that both meta-analysis methods generally produced better DE gene detection results than the five single study analyses, no matter PT, RIM_minP or RIM_BIC was used in single study analyses. Interestingly, although MD3_AMY generated more DE genes than that produced by meta-analysis methods (Fisher and maxP) using either RIM_minP or RIM_BIC (see Table 2), its pathway analysis performed worse than the two meta-analyses result and even worse than the other four single studies (Figure 5). This evidence may raise concern of quality in the MD3_AMY study that will need additional investigation. RIM_BIC and RIM_minP did not appear to generate more biologically validated results than PT, probably because of the currently limited knowledge of MDD neurobiology and the still largely inaccurate pathway information.

### Comparing fisher and maxP

In the literature, many microarray meta-analysis methods have been proposed and compared [11,34,38]. As was discussed in the method section, different methods have different strength for detecting different types of differentially expressed genes. In Li and Tseng [8], genes that are differentially expressed in all studies were termed as HS_A _type (hypothesis setting A) while genes differentially expressed in at least one study were called HS_B _type. Among the three methods compared in this paper, maxP were methods that detect HS_A _type DE genes, while Fisher's method detected HS_B _type DE genes. The result showed that the two meta-analysis methods detected different sets of DE genes, suggesting different algorithms and assumptions behind the methods. Additional file 1: Figure S3 shows heatmaps on genes detected by Fisher alone (Additional file 1: Figure 3A), maxP alone (Additional file 1: Figure S3B) or both (Additional file 1: Figure S3C). In Additional file 1: Figure S3A, majority of DE genes detected by Fisher but not by maxP were dominated by strong differential expression in one or two studies (many in MD3_AMY and some in MD2_ACC or MD3_ACC). Although Fisher's method has been popularly applied in the microarray meta-analysis literature, the result showed its weakness to be dominated by single strong signal studies that included potential false positives. For example, Fisher's method detected 810 DE genes among which 445 DE genes (about 55%) could also be detected in MD3_AMY) while only 169 (about 24%) among 683 DE genes detected by maxP method could be detected in MD3_AMY. On the other hand, maxP had better statistical power to detect many genes with weak DE evidence in all studies (Additional file 1: Figure 3B) that Fisher's method cannot detect. Conceptually, we were more interested in identifying genes differentially expressed across all studies through maxP.

### Post hoc analysis for confounding covariates

To evaluate the impact of covariates on the gene expression values and degree of confounding with disease effect, especially among DE genes, we counted the number of appearances of covariates in the RIM_minP model for DE genes detected by maxP method under FDR = 15%. We calculated the rank of each covariate in each study and computed rank averages of each covariate to indicate relative degree of frequency that a covariate impacted gene expression and confounded with disease effect (see Table 3). PMI (appeared in 13-20% RIM_minP models of 683 DE genes) and pH (appeared 12-30% in RIM_minP models) consistently had high rank, indicating that they seldom confounded and influenced the disease effect estimate. Suicide (appeared 22-50% in RIM_minP models), alcohol (appeared 29-54% in RIM_minP models) and antidepressant (appeared 17-53% RIM_models) were three factors that consistently ranked among the most influential factors. Finally, age (appeared 21-32% in RIM_minP models) was an intermediate confounding factor. The ranking of MD3_ACC and MD3_AMY was highly correlated (Spearman correlation = 0.89) and the correlation between rankings of MD1_ACC and MD1_AMY was also high (Spearman correlation = 0.54). The high within cohort correlations showed a cohort dependent structure and suggested that more studies may be needed to provide empirical evidence on the covariate impacts, particularly for the impact of antidepressant and suicide.

**Table 3:** Frequency of covariates appearing in RIM_minP model selection among 683 DE genes detected by maxP method under threshold FDR = 15%

	MD1_ACC	MD2_ACC	MD3_ACC	MD1_AMY	MD3_AMY	Rank average
Age	142(5)	213 (4)	205 (4)	173 (3)	218 (3)	3.6

Alcohol	299 (2)	279 (2)	221 (3)	368 (1)	195 (4)	2.4

Antidepressant	348 (1)	119 (6)	271 (2)	346 (2)	362 (1)	2.4

pH	208 (3)	150 (4)	116 (6)	108 (6)	86 (6)	5

PMI	93 (6)	120 (5)	141 (5)	133 (5)	120 (5)	5.2

Suicide	150 (4)	325 (1)	340 (1)	149 (4)	322 (2)	2.4

Rank is shown in parentheses and rank average of each covariate is calculated to indicate relative degree of frequency that a covariate impacts gene expressions and confounds with disease effect.

To further explore effects of covariates, we analysed a set of 9 genes that have been previously associated with MDD in the literature (see Table 1A and 1B). Intuitively, we expected that a covariate should be included in the model across studies more frequently than by random and effects of a covariate should have consistent differential expression direction across studies. We constructed two hypothesis testing using the co-appearing statistics *T_1 _*and concordant ratio statistics *R *described in the Method section and performed the tests on the set of 9 MDD-related genes. The result showed weak to marginal statistical significance of the first hypothesis (*p *= 0.397 based on RIM_minP and *p *= 0.011 based on RIM_BIC), suggesting covariates were consistently selected across studies by RIM_BIC but not RIM_minP. For the second hypothesis, tests for both 9 MDD gene list was statistically significant (*p *= 0.014 and 0.005). The effects sizes of selected confounding variables have concordant direction across studies. For example, age was found a confounding variable in gene NPY and TAC1 in three out of five studies and the effect sizes were all negative (in log scale). The moderate statistical significance is reasonable since the hypothesis tests were performed only on the nine selected genes. This result demonstrated that covariates overall impacted gene expression changes consistently and confounded with disease effects among the 9 MDD-related gene list.

### Simulation results

The results of simulations to evaluate Type I error control and statistical power of different methods are shown in Table 4. In Scenario I simulation, the effect of disease state X on gene expression Y was confounded by two out of ten clinical variables in Z (*Z *= (*z_1_*,...,*z_10_*); *z_1 _*and *z_2 _*are confounders while the other eight variables are not). The result showed that t-test had low statistical power due to the confounders (power = 0.679). FEM_ALL also had low power due to the inclusion of all ten clinical variables in the model while in fact, only two of the ten were effective confounders (power = 0.697). Both FEM models with variable selection performed well. FEM_BIC performed slightly better than FEM_minP (power = 0.746 versus 0.729). The type I errors for all methods were close to the nominal 5% rate, showing adequacy of the models and statistical inference. For Scenario II, all clinical variables were independent from the gene expression. Not surprisingly, t-test performed the best with statistical power 0.938. FEM_minP and FEM_BIC both had similar high power at 0.929 and 0.925. FEM_ALL forced all variables in the model and obtained a low statistical power at 0.85. From Scenario I and Scenario II simulation, FEM_BIC and FEM_minP performed well in both extreme cases, demonstrating its sensitivity and robustness. Scenario III examined a special situation that variables in Z impacted gene expression Y through disease state X. Similar to Scenario II, t-test performed the best in this situation since Z is not confounded (power = 0.938). Both FEM_BIC and FEM_minP had similar high power (power = 0.925 and 0.916) but FEM_ALL again had low power (power = 0.851). For scenario I, we simulated two confounding variables, z_1 _and z_2_, where z_1 _had a strong association with Y (gene expression) while z_2 _had a weaker association with Y. In Table 4, an average of 1.97 variables was selected by RIM_minP and 1.27 variables were selected by RIM_BIC. Among them, 0.97 (by RIM_minP) and 0.78 (by RIM_BIC) variables belong to the true confounding variables (*z_1_, z_2_*). The result showed effectiveness of RIM_minP and RIM_BIC in variable selection compared to paired t-test (always contains no confounding variable) and RIM_ALL (always include all ten variables in *Z*). Overall, the simulation results confirmed our findings in MDD data analysis that modelling confounding variables with variable selection had better sensitivity and robustness in DE gene detection.

**Table 4:** Evaluation of t-test, FEM_minP, FEM_BIC and FEM_ALL methods by simulations

	Type I error (s.e.)				Power (%) (s.e)				Number of DE genes (s.e)				# of variables in Z selected	
**Scenario**	**A**	**B**	**C**	**D**	**A**	**B**	**C**	**D**	**A**	**B**	**C**	**D**	**B**	**C**

I	0.051 (.001)	0.046 (.001)	0.049 (.001)	0.051 (.001)	67.9 (.006)	72.9 (.007)	74.6 (.006)	69.7 (.006)	12.5 (1.03)	20.4 (1.11)	23.3 (1.04)	17.6 (1.09)	0.97/1.97*	0.78/1.21*

II	0.051 (.001)	0.052 (.001)	0.050 (.001)	0.051 (.001)	93.8 (.003)	92.9 (.003)	92.5 (.003)	85.0 (.005)	73.4 (.85)	73.0 (.92)	69.8 (.96)	49.7 (1.37)	1.7	0.59

III	0.051 (.001)	0.053 (.001)	0.051 (.001)	0.051 (.001)	93.8 (.003)	92.5 (.004)	91.6 (.004)	85.1 (.005)	71.8 (.93)	68.3 (.94)	66.5 (.88)	45.8 (1.05)	1.8	0.6

*The denominator showed average number of variables in Z selected. The numerator showed average number of selected variables that belong to the true confounders (*z_1_, z_2_*).

The average of type I errors, average of statistical powers, and average number of detected DE genes by each method are shown. Standard errors are shown in parentheses. In the last two columns, the average numbers of confounding variables selected by FEM_minP and FEM_BIC are shown.

A: t-test, B: FEM_minP, C: FEM_BIC, D: FEM_ALL

## Discussion

In this paper, we described a statistical approach adjusted for confounding variables (i.e. a random intercept model with variable selection), to tackle weak signal expression profiles that have small sample size, case-control paired design and confounding covariates in each study. The results showed increased statistical power from confounding variable adjustment, paired design modelling and meta-analysis in this genomic setting and improved biological findings and interpretations have been discovered in MDD neurobiology. Pathway analysis and post hoc analysis of variable selection revealed insightful biological conclusions. Simulations under three correlation structures were performed to verify improved performance of the proposed model. In the literature, most psychiatric disease related microarray studies of similar design either ignored the clinical variables or applied simple linear regression to include all variables in the model. Our simulations clearly show limits to those two approaches. Our approach systematically considers the critical elements in the data structure in order to obtain more accurate DE gene and pathway detection. The framework is general and can be applied to microarray meta-analysis of other complex diseases with similar data structure. Specifically, this approach will be of great use in human post-mortem studies of the brain, where confounding factors are intrinsic (1) to the nature of the cohorts (demographic parameters), (2) to their method of collection (post-mortem interval) and (3) to the illness per se (clinical heterogeneity). Since dilution of expression signal is likely to occur in complex tissue such as the brain, DE genes often show small and weak effects and eliminating the effects of confounding factors is critical to detect disease associated markers.

In the variable selection of the RIM model, we tested both BIC and minP approaches. The real data analysis showed that minP seemed to identify more DE genes in the MDD example while simulations showed similar performance and statistical power of the two methods. Another potential alternative is to apply popular regularization and shrinkage methods, such as Lasso or ridge regression, in the variable selection. A prohibitive down-side of such approaches is its expensive computational load for genome-wide analysis, particularly in the estimation of the tuning parameters. In our analysis, BIC and minP procedures were limited to up to two covariates in the model, which balanced well in biological interpretation and computation feasibility.

The goal of this study was to determine optimal analytical approaches for complex datasets with multiple putative confounding variables. For this purpose, we focused on datasets produced by a single laboratory, in order to avoid additional confounding factors due to differences in laboratory protocols, brain bank collection, tissue treatment and sample handling. Now that we have established such analytical guidelines, the next step will be to increase the scope of meta-analyses by including additional datasets that are progressively made available in the literature. However, as expected, this also comes with added variability, which necessitates the development of complementary mathematical tools. For instance, we have designed a data-driven "meta-QC" quality control approach to rigorously assess the quality of microarray datasets to be combined [39]. The quality control test is critical to assess whether the inclusion of additional datasets will increase the analytical power, or be detrimental to the meta-analysis. Finally, as briefly elucidated in this report, mechanisms underlying neurological and neuropsychiatric disorders are likely to involve a distributed sets of brain regions linked in functional neural networks. The detection of molecular pathologies associated with those disorders will thus also critically depend on *a priori *hypotheses for converging or opposing effects in selected brain regions, for the presence (or not) of control brain regions. For instance, genetic risk factors may be hypothesized to similarly affect biological pathways across brain regions, while compensatory mechanisms leading to pathological dysfunction may display regional specificity, depending on the respective activation or inhibition of different components of neural networks. Hence, the biological impact of the studies performed here will be investigated, validated and discussed more in-depth elsewhere.

The studies combined in this paper have significant cohort features that may introduce significant heterogeneity. The five studies came from three distinct cohorts (MD1, MD2 and MD3), different sexes (male and female), array platforms (Affymetrix and Illumina) and brain regions (ACC and AMY). Future research is currently pursued to decipher such study-specific features. A future direction is to collect more studies and apply meta-regression techniques to identify sex-specific or brain-region-specific genes in a unified meta-analysis.

## Competing interests

The authors declare that they have no competing interests.

## Authors' contributions

The motivating data were generated from ES's lab. GCT and ES conceived the general concept and analytical approaches. XW, ES and GCT brainstormed the detailed framework, method and algorithms. XW performed most of the programming and data analysis. YL, XW and GCT discussed and performed the simulation. XW, CS and GCT discussed and performed the p-value correction of the RIM_minP and RIM_BIC methods. All authors read and approved the final manuscript.

## Supplementary Material

Additional file 1 **Supplement material**. Click here for file
