# Supplementary material for: Detecting disease-associated genes with confounding variable adjustment and the impact on genomic meta-analysis: With application to major depressive disorder
Source: BMC Bioinformatics. 2012 Mar 29;13:52. doi: 10.1186/1471-2105-13-52 (PMC3342232; doi:10.1186/1471-2105-13-52)

Supplement Material

Manuscript title: Detecting disease-associated genes with confounding variable adjustment and the impact on genomic meta-analysis: with application to major depressive disorder.
By Xingbin Wang, Yan Lin, Chi Song, Etienne Sibille, George C. Tseng

Part I. Permutation analysis to correct p-values in RIM_minP and FEM_minP.

The disease effect p-values obtained from the best model selected by RIM_minP or RIM_BIC are biased from variable selection. Below permutation analysis generates null hypothesis and produces correct inference of type I error (p-value) control.

Step1: For each given gene g, fit all possible RIM (FEM) models that include at most two (0,1,or 2) clinical variables. In each model, calculate the p-values associated with the disease effect using likelihood ratio test (LRT) under the null hypothesis $H_o: \beta_{g0} = 0$.

Step2: Select the resulting RIM_minP (FEM_minP) model that has the smallest p-value among all models. Denote by the resulting minimum p-value as $p_g^{(o)}$.

Step3 Permute the labels of disease and control within each pair (if the experiments were not pair-designed, permute the labels of disease and control among all the samples) for B times. In the bth permutation, repeat step1-2 to get minimum p-value for null distribution, $p_g^{(b)}$ $(1 \le b \le B,\ 1 \le g \le G)$;

Step4: The corrected p-value for gene g is calculated as $p_g = \frac{\sum_{g'=1}^{G}\sum_{b=1}^{B} I\left(p_{g'}^{(b)} \le p_g^{(o)}\right)}{G \cdot B}$, where $I(.)$ is an indicator function, which takes value one when the statement is true and zero otherwise.

Remark:1
Similarly, the above procedures can be used to correct the p-values associated with the RIM_BIC (FEM_BIC) models. Note that BIC is used to choose the RIM_BIC (FEM_BIC) models in step 2-3.
Remark:2
To account for dependency structure of studies using the same cohorts (MD1_ACC and MD1_AMY, MD3_ACC and MD3_AMY), we kept the same permutation in step 3 for the two pairs of studies.

Part II. Pathway analysis –KS test

For gene enrichment analysis, we performed Kolmogorove-Smirnov (KS) test, which was widely used in gene enrichment analysis because it is sensitive to differences in both location and shape of the empirical cumulative distribution functions of the two samples. Specifically, the p-values calculated from individual analyses or meta-analyises for assessing the DE genes are classified into two categories, in the pathway (P) and out of pathway (PC). Let $p_{(1)}, p_{(2)}, \ldots, p_{(n)}$ and $\tilde{p}_{(1)}, \tilde{p}_{(2)}, \ldots, \tilde{p}_{(m)}$ denote the order statistics of the p-values in P and PC, respectively. The corresponding empirical distribution functions, $\hat{F}_P(x)$ and $\hat{F}_{P^C}(x)$ for P and PC can be defined as:

$$\hat{F}_P(x) = \begin{cases} 0, x < p_{(1)} \\ \frac{k}{n}, p_{(k)} \le x < p_{(k+1)}\ k = 1, 2, ..., n-1 \\ 1, x \ge p_{(n)} \end{cases}$$

and

$$\hat{F}_{P^C}(x) = \begin{cases} 0, x < p_{(1)} \\ \frac{k}{m}, p_{(k)} \le x < p_{(k+1)}\ k = 1, 2, ..., m-1 \\ 1, x \ge p_{(m)} \end{cases}.$$

Let FP and FPC denote the population distribution for P and PC, respectively. The one-sided two sample KS test can be defined based on the formula: $T_{KS} = \max_x [F_P(x) - F_{P^C}(x)]$, where the null hypothesis and the alternative hypothesis are :

$H_0 : F_P(x) = F_{P^C}(x)$ for all x

$H_a : F_P(x) \ge F_{P^C}(x)$ for all x

$\& F_P(x) > F_{P^C}(x)$ for some x

Under the null hypothesis, the rejection region has the form of $T_{KS} > C_\alpha$ at level of α. Rejection of H_0 means that P is stochastically less than PC (the CDF of P lies above and hence to the left of that for PC). In nother words, the p-values of genes in the pathway P are stochastically less than the p-values of genes outside of pathway PC. Small p-value associated with KS test indicates a good performance of the methods.

Part III. Procedure to test consistency of covariate effects in detected DE genes

We denote by e_{glk} the effect of covariate $X_l (1 \le l \le L)$ on the gene expression of gene g in the kth study if the covariate is selected by RIM_minP model selection. When covariate X_l is not selected by RIM_minP, e_{glk} is not defined. Define

$$C_{gl}(i,j) = \begin{cases} 1, \text{ if } e_{gli} \cdot e_{glj} > 0 \\ -1, \text{ if } e_{gli} \cdot e_{glj} < 0 \\ 0, \text{ if either } e_{gli} \text{ or } e_{glj} \text{ is not defined} \end{cases}$$

$C_{gl}(i,j)$ takes value 1 if covariate X_l $(1 \le l \le L)$ appears in the RIM_minP models in study i and j, and both effects have the same direction (both positive or both negative). In this situation, the effects of X_l $(1 \le l \le L)$ in study i and j are consistent. On the contrary, $C_{gl}(i,j)$ takes values -1 when covariate X_l $(1 \le l \le L)$ appears in the RIM_minP models in study i and j, and have discordant effect sizes. When the covariate X_l $(1 \le l \le L)$ does not appear in the RIM_minP model of either study i or j, $C_{gl}(i,j)$ takes value 0. To test whether the covariates are selected by common covariates across studies more frequently than random, we calculate the total number of times a covariate is selected by RIM_minP among all pairs of studies for a given gene set G' and denote as test statistics T_1 below:

$$T_1(G') = \sum_{g \in G'} \sum_{l=1}^{L} \sum_{1 \le i < j \le K} \left| C_{gl}(i,j) \right|$$

To test the concordance of covariate effects across studies, we count only the concordant cases in T_1:

$$T_2(G') = \sum_{g \in G'} \sum_{l=1}^{L} \sum_{1 \le i < j \le K} I\left(C_{gl}(i,j) = 1\right)$$

The concordance rate is then defined as $R(G') = T_2(G')/T_1(G')$. To test whether $T_1(G')$ and $R(G')$ is larger than obtained by random with statistical significance. Permutation analysis below is performed:

Step1: Given a table of gene set G', we calculate the observed statistics of $T_1(G')$ and $R(G')$ and denote them as $T_1^{(o)}$ and $R^{(o)}$, respectively;

Step2: Randomly permute the observed (0,1,-1) values across clinical variables for a given gene and a given study for B times. For each permuted data, calculate the $T_1(G')$ and $R(G')$ similarly to obtained $T_1^{(b)}$ and $R^{(b)}$.

Step3: Calculate the p-values associated with $T_1(G')$ and $R(G')$ as $p_{T_1} = \frac{\sum_{b=1}^{B} I(T_1^{(b)} \ge T_1^{(o)})}{B}$ and $p_R = \frac{\sum_{b=1}^{B} I(R^{(b)} \ge R^{(o)})}{B}$.

Part IV. Simulation scheme of three correlation structures in Scenario I, II and III.

(X: disease state; Y: gene expression; Z: ten potential confounding variables)

Scenarios	Simulation models	Detailed simulation procedures
Scenario I<br>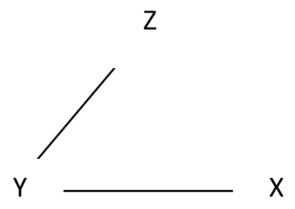 	$Z_1, \ldots, Z_{10} \sim BIN(1,0.5)$<br>Y is linked to X , Z_1 and Z_2 by equation:<br>$Y = \beta_0 + \beta_1 * X + \beta_2 * Z_1 + \beta_3 * Z_2 + \varepsilon$,<br>Where $\varepsilon \sim N(0, \sigma^2)$	Step 1: Simulate $Z_1, \ldots, Z_{10}$ i.i.d. from $BIN(1,0.5)$<br>Step 2: Simulate microarray data<br>• 100 DE genes:<br>Cases: (the first 25 samples, X=1)<br>Y=$\beta_0 + \beta_1 + \beta_2 * Z_1 + \beta_3 * Z_2 + \varepsilon$,<br>$\varepsilon \sim N(0, \sigma^2)$<br>Controls: (the last 25 samples, X=0)<br>Y=$\beta_0 + \beta_2 * Z_1 + \beta_3 * Z_2 + \varepsilon$,<br>$\varepsilon \sim N(0, \sigma^2)$<br>• 900 Non-DE genes Y=$N(\beta_0, \sigma^2)$ for all 50 samples.<br><br>Paraeter setting: $\beta_0 = 0$, $\beta_1 = 0.8$, $\beta_2 = 0.6$, $\beta_3 = -0.3$, $\sigma = 1$.
Scenario II<br>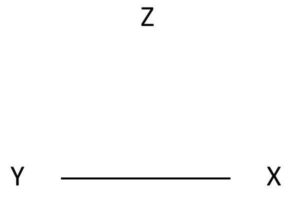 	$Z_1, \ldots, Z_{10} \sim BIN(1,0.5)$<br>Y is linked to X alone by equation:<br>$Y = \beta_0 + \beta_1 * X + \varepsilon$,<br>Where $\varepsilon \sim N(0, \sigma^2)$	Step 1: Simulate $Z_1, \ldots, Z_{10}$ i.i.d. from $BIN(1,0.5)$<br>Step 2: Simulate microarray data<br>• 100 DE genes:<br>Cases: (the first 25 samples, X=1)<br>Y=$\beta_0 + \beta_1 X + \varepsilon$, $\varepsilon \sim N(0, \sigma^2)$<br>Controls: (the last 25 samples, X=0)<br>Y=$\beta_0 + \varepsilon$, $\varepsilon \sim N(0, \sigma^2)$<br>• 900 Non-DE genes<br>Y=$N(\beta_0, \sigma^2)$ for all 50 samples.<br><br>Parameter setting: $\beta_0 = 0$, $\beta_1 = 1.5$, $\sigma = 1$.
Scenario III<br>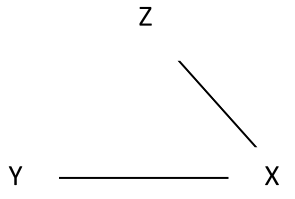 	Z_1 and X are linked to each other by equation:<br>$logit(\Pr(Z_1 = 1)) = \gamma_0 + \gamma_1 * X$<br>$Z_2, \ldots, Z_{10} \sim BIN(1,0.5)$<br>Y is linked to X and Z by equation:<br>$Y = \beta_0 + \beta_1 * X + \varepsilon$,<br>Where $\varepsilon \sim N(0, \sigma^2)$	Step 1: Simulate $Z_2, \ldots, Z_{10}$ i.i.d. from $BIN(1,0.5)$<br>Step 2:Simulate $Z_1$by<br>$Z_1\|_X \sim BIN(1, \frac{1}{1 + e^{-\gamma_0 - \gamma_1 \cdot X}})$<br>Step 3: Simulate microarray data<br>• 100 DE genes:<br>Cases: (the first 25 samples, X=1)<br>Y=$\beta_0 + \beta_1 + \varepsilon$, $\varepsilon \sim N(0, \sigma^2)$<br>Controls: (the last 25 samples, X=0)<br>Y=$\beta_0 + \varepsilon$, $\varepsilon \sim N(0, \sigma^2)$<br>• 900 Non-DE genes<br>Y=$N(\beta_0, \sigma^2)$ for all 50 samples.<br><br>Parameter setting: $\gamma_0 = 0, \gamma_1 = 2, \beta_0 = 0$, $\beta_1 = 1$, $\sigma = 1$.

Supplement Table 1. Data description of five MDD microarray studies

Study name	Gender	Brain region	Sample size	Array platform
MD1_ACC	Male	ACC	32 (16 pairs)	Affymetrix
MD2_ACC	Male	ACC	20 (10 pairs)	Illumina
MD3_ACC	Female	ACC	50 (25 pairs)	Illumina
MD1_AMY	Male	AMY	28 (14 pairs)	Affymetrix
MD3_AMY	Female	AMY	42 (21 pairs)	Illumina

Cohort MD1 was described in Sibille et al (2009), A molecular signature of depression in the amygdala. Am.J.Psychiatry, 166, 1011-1024. MD2 and MD3 cohorts will be described in details elsewhere.

Supplement Table 2. Pearson correlation between covariates in three MDD cohorts (collinearity evaluation)

	Age	Alcohol	Antidep	Suicide	pH	PMI
Age	---	(-0.05, 0.34, 0)*	(0.15, 0.14, 0.04)	(0.02, -0.26, 0)	(-0.12, -0.01, -0.04)	(-0.19, -0.17, 0.37)
Alcohol	(-0.05, 0.34, 0)	---	(-0.21, 0.63, 0.28)	(0.41, 0.15, 0.22)	(0.09, 0.22, -0.08)	(-0.02, -0.29, -0.04)
Antidep	(0.15, 0.14, 0.04)	(-0.21, 0.63, 0.28)	---	(0.31, 0.19,0.22)	(0.18, 0.36,-0.21)	(-0.13, -0.35, -0.18)
Suicide	(0.02, -0.26, 0)	(0.41, 0.15, 0.22)	(0.31, 0.19,0.22)	---	(0.19, -0.3, 0.06)	(-0.17, -0.38, -0.02)
pH	(-0.12, -0.01, -0.04)	(0.09, 0.22, -0.08)	(0.18, 0.36,-0.21)	(0.19, -0.3, 0.06)	---	(0.41, -0.03, -0.03)
PMI	(-0.19, -0.17, 0.37)	(-0.02, -0.29, -0.04)	(-0.13, -0.35, -0.18)	(-0.17, -0.38, -0.02)	(0.41, -0.03, -0.03)	---

* The three numbers in parentheses are Pearson correlation between two covariates in MD1, MD2 and MD3 patient cohorts.

Supplement Table 3. The number of significant interaction terms between disease state and covariates in FEM and RIM model under FDR=5%.

		FEM					RIM				
		MD1_ACC	MD2_ACC	MD3_ACC	MD1_AMY	MD3_AMY	MD1_ACC	MD2_ACC	MD3_ACC	MD1_AMY	MD3_AMY
FDR=0.05	Age	0	0	0	0	0	37	1	0	0	0
	pH	0	0	0	0	0	0	3	0	0	0
	PMI	0	0	0	0	0	0	1	0	0	0

Supplement Figure 1. A diagram for data preprocessing, gene matching and gene filtering.

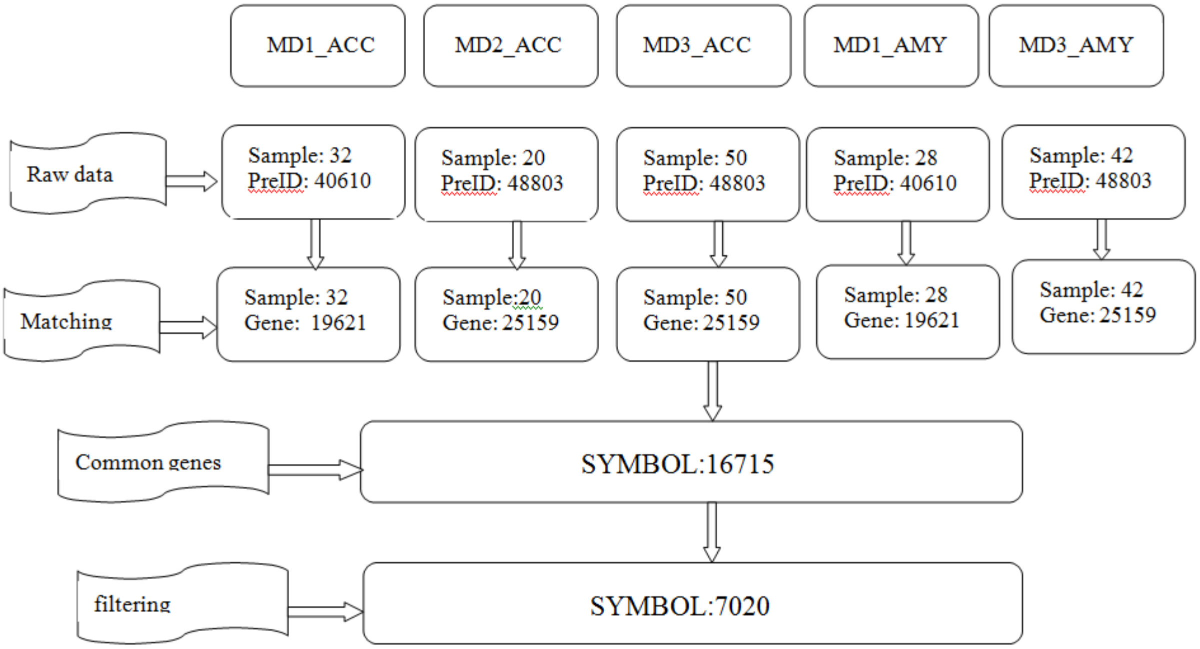

Supplement Figure 2. Null distributions of disease effect p-value in model selection (upper: RIM_minP; lower: RIM_BIC) simulated from permutation analysis in the five MDD studies. The result shows bias (deviation from uniform distribution) caused by variable selection.

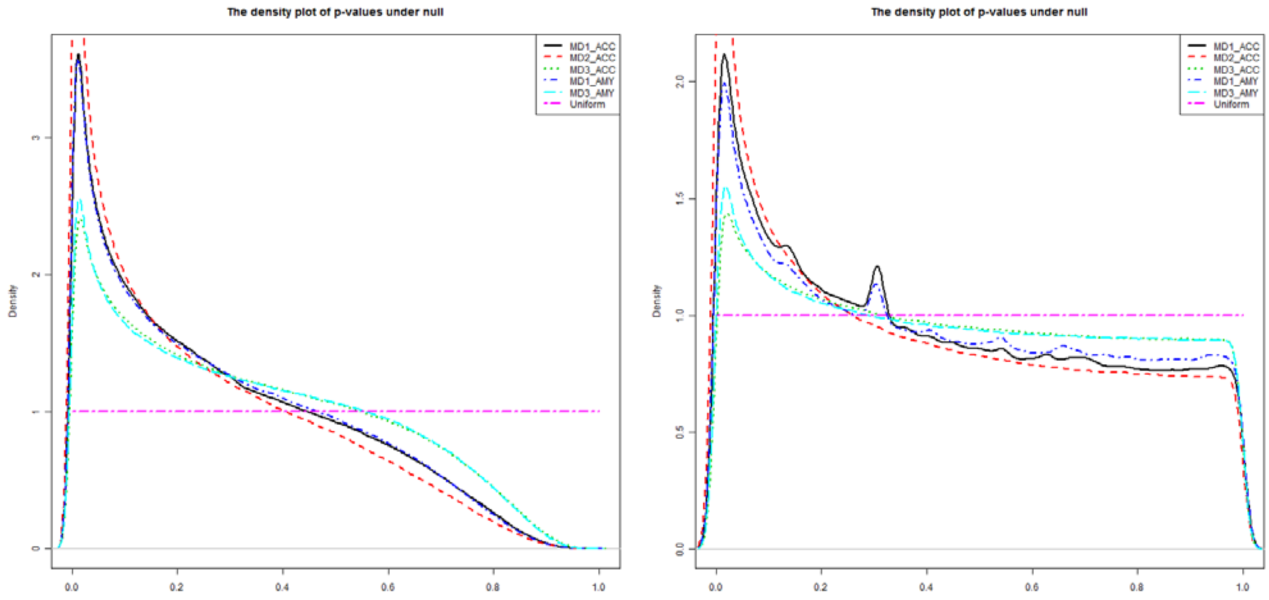

Supplement Figure 3. Heatmap of minus log10-transformed p-values obtained from all five studies and meta-analysis for detecting DE genes under FDR=15%. Red indicates small p-values and green indicates large p-values. (A) DE genes detected by Fisher's method but not by maxP method; (B) DE genes detected by maxP but not by Fisher's method; (C) DE genes detected by both Fisher and maxP method.

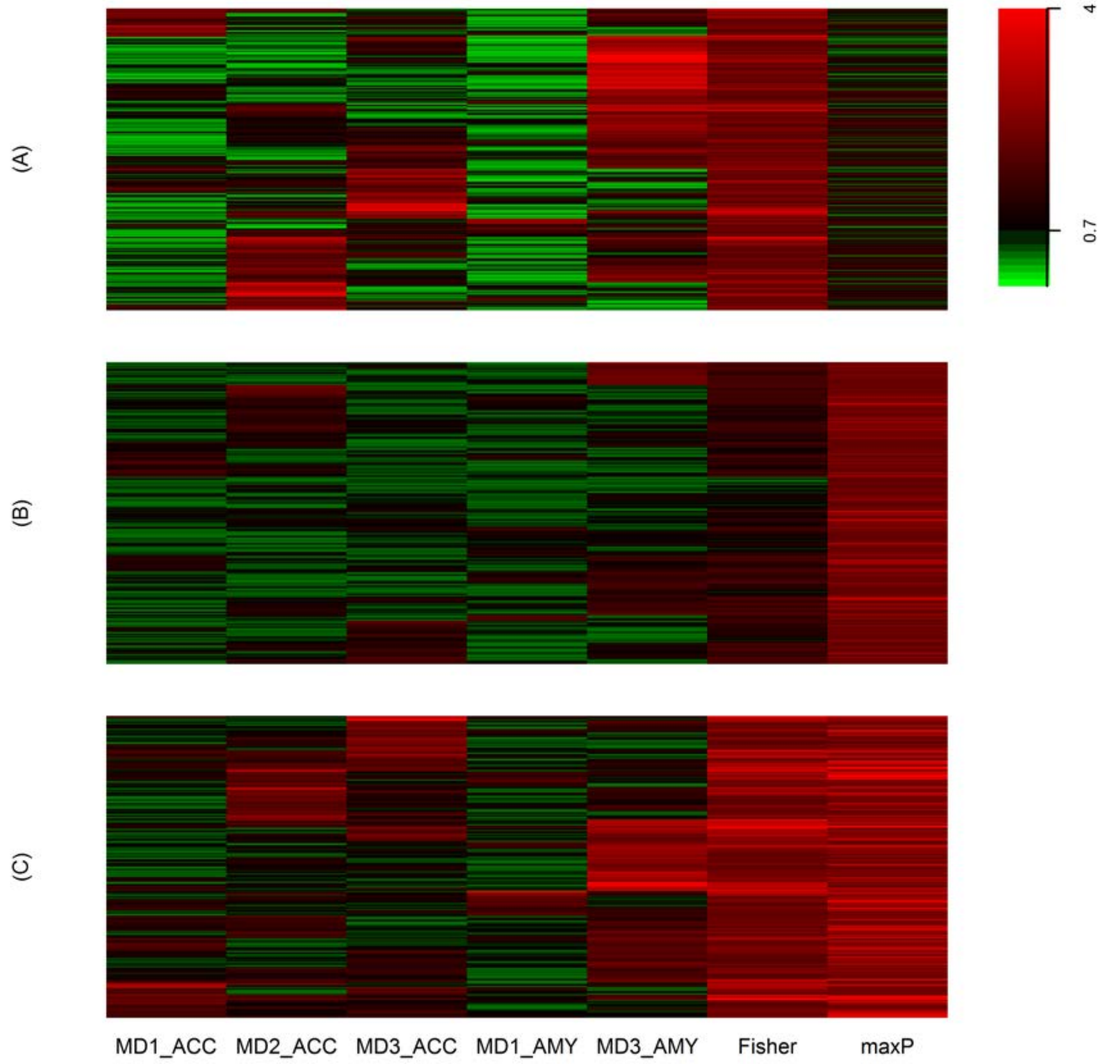

Supplement: Additional file 1 — Supplement material. [file 1471-2105-13-52-S1.PDF]
